# Temporal Change Rate in Sound Velocity Caused by Ultrasonic Heating for Evaluation of Steatotic Liver

**DOI:** 10.3390/biology14111585

**Published:** 2025-11-13

**Authors:** Machi Itsubo, Yume Kobayashi, Masaki Yamamoto, Shinji Takayanagi, Iwaki Akiyama

**Affiliations:** 1 Department of Biomedical Sciences and Informatics, Faculty of Life and Medical Sciences, Doshisha University, Kyotanabe 610-0394, Japaniakiyama@mail.doshisha.ac.jp (I.A.); 2 Department of Medical Life Systems, Faculty of Life and Medical Sciences, Doshisha University, Kyotanabe 610-0394, Japan

**Keywords:** tissue characterization, change rate in sound velocity, ultrasonic heating, steatotic liver, thermos-physical property

## Abstract

Steatotic liver diseases are increasing globally, with metabolic dysfunction-associated steatohepatitis potentially causing irreversible fibrosis progression. This study focuses on an ultrasonic diagnostic method for steatotic liver disease based on temperature dependence of sound velocity for tissue characterization. Since the temperature coefficient of sound velocity in liver is expected to decrease with increasing lipid accumulation, the temperature coefficient of sound velocity in tissue-mimicking material as a function of glycerol concentration was measured. It decreased as glycerol concentration increased, changing from positive to negative value at 37.5% glycerol concentration. Change rates in sound velocity by ultrasonic heating were then measured in vitro on liver left lobes of mice with steatotic liver induced by choline-deficient, L-amino acid-defined, and high-fat diet. There were positive values in the control group, whereas there were negative values in the steatotic liver group. In vivo measurements of mouse livers using an electrocardiogram-synchronized system showed similar results, with positive values in the control group and negative values in the steatotic liver group. Thermophysical properties can determine whether the liver is normal or steatotic. However, to estimate the lipid accumulation rate from the change rate in sound velocity, it is necessary to reduce the measurement variation.

## Simple Summary

The prevalence of steatotic liver diseases is on the rise globally, with metabolic dysfunction-associated steatohepatitis being a significant concern due to its potential to lead to irreversible fibrosis. This study investigated a noninvasive method of diagnosing steatotic liver diseases using ultrasound. It is known that the sound velocity of fat tissue decreases with heating, whereas that of non-fat tissue increases with heating. Therefore, the change rates in sound velocity were measured in vitro and in vivo on mouse livers from the control and steatotic liver groups by ultrasonic heating within safety standards. In both in vitro and in vivo measurements, there were positive values in the control group and negative values in the steatotic liver group. The results of these change rates in sound velocity indicated that it is possible to determine whether the liver is normal or steatotic.

## 1. Introduction

Steatotic liver diseases are increasing worldwide [1,2,3,4,5], and among them, metabolic dysfunction-associated steatohepatitis may cause irreversible progression of fibrosis, and follow-up is important clinically [6,7,8,9,10]. This study concentrates on measurement of temperature dependence of sound velocity for biological tissues as an approach of tissue characterization [11,12,13,14,15]. When biological tissue is exposed to ultrasound, the absorption of acoustic energy generates heat within the tissue, resulting in an increase in tissue temperature. The temperature rise depends on the ultrasound intensity, specific heat, thermal diffusion coefficient, attenuation coefficient, and exposure time. It has been reported that the temperature coefficients are negative values for fat tissues and positive values for non-fat tissues [15,16,17,18,19,20,21,22]. The temperature coefficients for porcine muscle and fat tissues were reported as 1.2 m/s/°C and −3.1 m/s/°C, respectively [20]. The temperature coefficients for hepatocellular carcinoma (HCC) and normal liver of rats are 1.59 ± 0.04 and 1.07 ± 0.02, respectively [23]. The change rate in sound velocity due to the ultrasonic heating of the porcine muscle and fat tissues was measured in vitro using a concave transducer in which the heating transducer and the sound velocity measurement transducer are coaxially integrated [20]. The results showed a positive rate of sound velocity change in the muscle tissue and a negative rate of sound velocity change in the fat tissue, as well as the temperature coefficients of sound velocity. The estimated temperature rise during the measurement was 0.36 °C and 1.1 °C for the muscle and fat tissue, respectively, when the ultrasonic heating time was shortened to one second or less. The temperature increase by the ultrasonic heating in this measurement method satisfies the in vivo safety standard of 1.5 °C or less. Therefore, the rate of change in sound velocity due to ultrasonic heating is expected to be a physical property for the ultrasound tissue characterization.

In this study, mouse models of steatotic liver disease were used to evaluate the change rates in sound velocity. Those of the left lobes of normal liver and steatotic liver were measured in vitro before and after ultrasonic heating. As a result, they were positive in the control group, whereas they were negative in the steatotic liver disease group. Then, the measurement system synchronized with the heartbeat of a mouse was prepared to measure the change rates of sound velocity in vivo. As with the in vitro measurements, they were positive in the control group, whereas they were negative in the steatotic liver disease group. Furthermore, the change rates of sound velocity were suggested to increase negatively as the rate of lipid accumulation increased. It is shown that the change rate in sound velocity due to the ultrasonic heating has a potential to evaluate steatotic liver disease in the clinical diagnosis.

## 2. Materials and Methods

### 2.1. Measurement of Temperature Coefficient of Sound Velocity in Tissue-Mimicking Material (TMM) Phantoms

The temperature coefficients of sound velocity were measured for TMM phantoms with glycerol solution by varying the concentration. TMM is defined as IEC 60601-2-37 standard [24]. The concentration of four phantoms were 20.00, 27.00, 34.00, and 43.00%, respectively.

**Figure 1:**
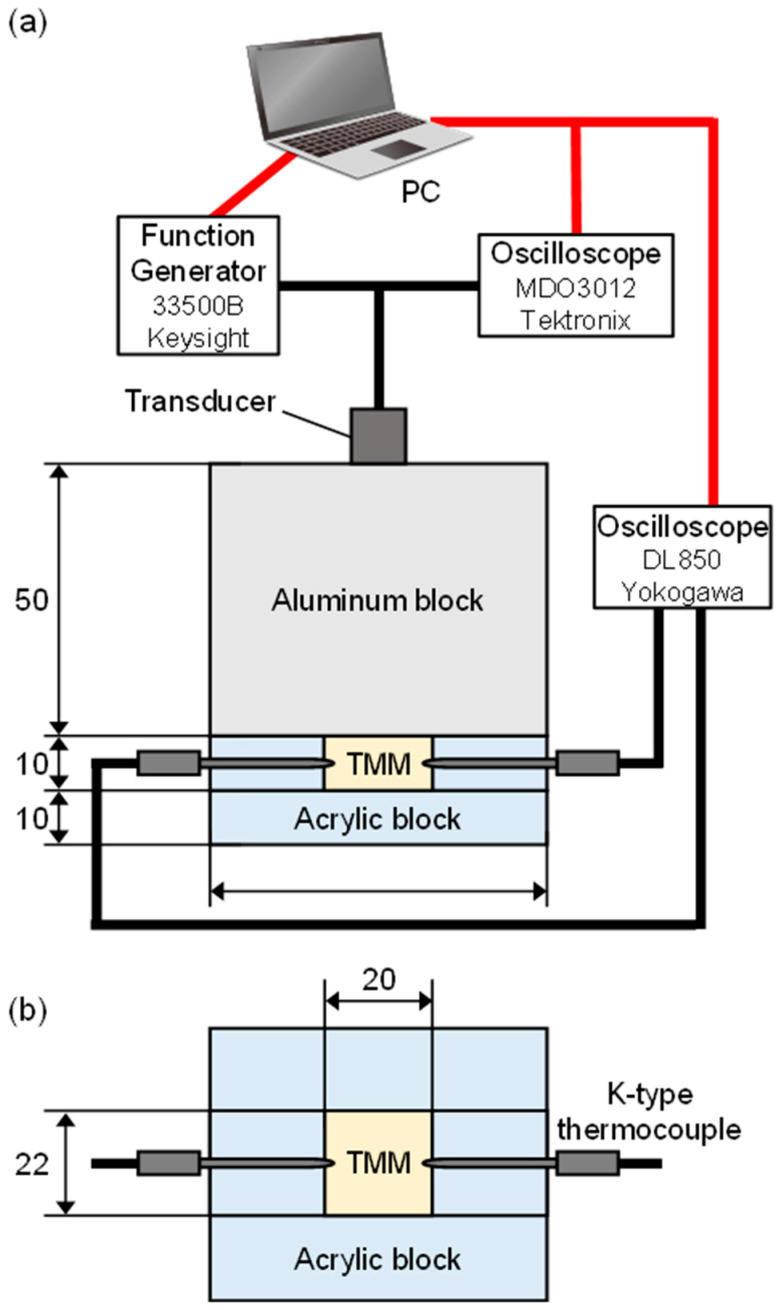
Experiment system to measure the temperature coefficient of sound velocity in (**a**) cross-sectional side view and (**b**) cross-sectional top view. Temperature of TMM was measured at two points by two K-type thermocouples. The red and black lines represent digital cables and analog cables, respectively.

The experimental system for the measurement of sound velocity of the TMM samples is shown in Figure 1 [20,21]. To raise the TMM temperature from room temperature to approximately 30 °C, experiments were carried out in an incubator as shown in Figure 1a. The sound velocity in the TMM sample was measured by the pulse-echo method. The sample was exposed to ultrasonic wave of frequency of 5.0 MHz by driving a planar shape transducer (B5K10I, JAPAN PROBE, Yokohama, Japan) with a diameter of 10 mm at 10 V_P-P_ and 1 cycle of sinusoidal wave. A signal generator (33500B, Keysight Technologies, Santa Rosa, CA, USA) was used for transmissions of ultrasonic waves. The sample was exposed to ultrasound by passing through a 50 mm thick aluminum block to avoid the multiple reflection error. The transducer received two reflected waves returned from the front boundary between the sample and the aluminum block and the back boundary between the sample and the agar-gel block. The received echo signals were digitized using an oscilloscope (MDO3012, Tektronix, Beaverton, OR, USA) at 8 bits and 250 MS/s.

The sound velocity *c* of the sample is given by

(1) $$c=\frac{2d}{t},$$

using the sample thickness *d* and the time *t* for propagation in the sample. *t* was obtained from the time difference between the two echo signals. The temperatures inside the sample were measured by thermocouples (K-type, CHINO, Tokyo, Japan) and an oscilloscope (DL850E, Yokogawa Electric Corp., Tokyo, Japan), as shown in Figure 1b. The temperature coefficients of sound velocity were calculated by a linear approximation of the velocity versus temperature plot.

### 2.2. In Vitro Measurement of Change Rate in Sound Velocity

When biological tissue is exposed to ultrasound, the ultrasonic heating effect causes the sound velocity to change over time according to its temperature coefficient, as described in the previous section. Since the phase of the echo changes when the sound velocity changes, the rate of change in the sound velocity can be estimated by comparing the phase of the echoes before and after ultrasonic heating. The rate of change in sound velocity is proportional to the tissue’s inherent temperature coefficient of sound velocity. Thus, the change rate in sound velocity can be a parameter for tissue characterization.

As a dietary model for inducing steatotic liver disease in a short period of time, three 6-week-old C57BL/6 mice and three 12- to 14-week-old C57BL/6 mice were fasted for eighteen hours and then administered a choline-deficient, L-amino acid-defined, and high-fat diet (CDAHFD) and 10% sucrose water for three days. As control groups, three 6-week-old C57BL/6 mice and three 12- to 14-week-old C57BL/6 mice were fed a standard diet and water. Their livers were removed under general anesthesia, and their left lobes were kept in organ storage solution (Belzer UW^®^ Cold Storage Solution, Bridge to Life, Northbrook, IL, USA). To remove air bubbles in the left lobe, they were placed in a desiccator while in the organ storage solution and degassed with a vacuum pump for one hour. The change rates of sound velocity due to ultrasonic heating in these left lobes were measured. Histopathological analyses of them were also performed after the measurements.

This study was approved by the Doshisha University Animal Experiment Committee. All experiments were conducted in accordance with the Doshisha University Regulations on the Conduct of Animal Experiments and Related Activities.

**Figure 2:**
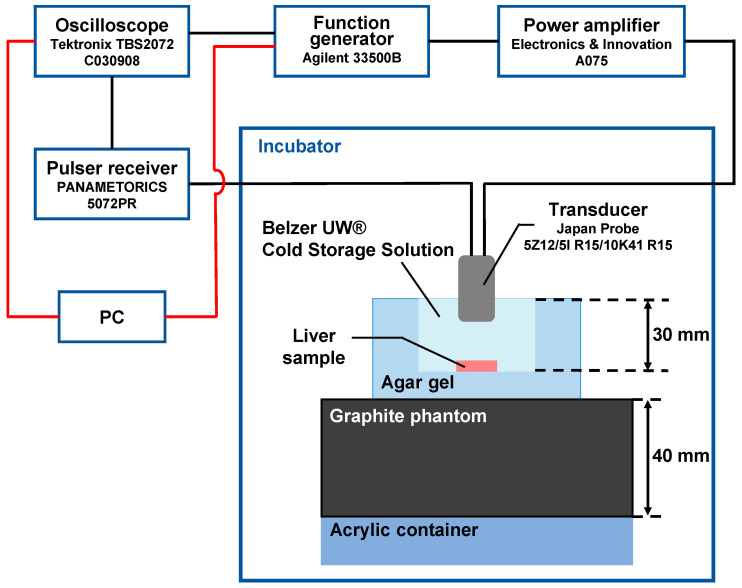
Experiment system for in vitro measurement of change rate in sound velocity of the liver sample due to the ultrasonic heating. The red and black lines represent digital cables and analog cables, respectively.

Figure 2 shows the experimental system for in vitro measurement of the change rate in sound velocity of the sample caused by ultrasonic heating. The temperature was controlled in an incubator at 20 °C before ultrasonic heating. The left lobe of the mouse liver was used as a sample. The sample was immersed in an agar-gel dish filled with the organ storage solution. The dish was placed on an agar-gel block with graphite powder dispersed in it, since it absorbed the ultrasonic energy of reflected waves generated at the boundary between the acrylic block and agar gel block.

In this experiment, a concave circular transducer with a diameter of 4 mm and a center frequency of 10 MHz was used for measurement by a pulse-echo method [20,25]. A concave ring-shaped transducer with an inner diameter of 5 mm, an outer diameter of 12 mm, and a center frequency of 5 MHz was used for ultrasonic heating. The focal length of both of transducers was 15.5 mm. The transducer was aligned so that the surface of the sample was adjusted to bring it into the focus. A waveform generator (33500B, Agilent Technologies, Santa Clara, CA, USA) and a power amplifier (A075, Electronics and Innovations, Rochester, NY, USA) were connected to the ring-shaped transducer to transmit the heating ultrasonic waves. The sample was exposed to ultrasound of 0.81, 1.2, and 1.6 MPa in negative sound pressures. The pulse-echoes from the specimen were received by the circular transducer which was connected to a pulsar receiver (5072PR, Panametrics, Billerica, MA, USA). The specimen was exposed to pulsed ultrasonic waves at 1 ms of pulse repetition time. The echo signals from the left lobe were digitized with an oscilloscope (TBS2072, Tektronix, Beaverton, OR, USA) at a quantization bit rate of 8 bits and a sampling frequency of 250 MHz. Each sample was successively heated for 10 ms and repeated 10 times at 2 s intervals.

When the temperature of the sample increases due to ultrasonic heating, the sound velocity changes. Therefore, when a pulse wave is transmitted from the transducer for sound velocity measurement and the echo signal from inside the specimen is observed, the arrival time of the echo signal is shifted in proportion to the change in sound velocity after ultrasonic heating. When the temperature rises differently at two points inside the specimen, a time gate is applied around the echo time from each point, and the echo time shifts due to ultrasonic heating within the gate are calculated. The difference between the two echo time shifts is Δ*τ*, and the time difference between the two gated points is Δ*t*. When the sound velocity change Δ*c*(*x*) at position *x* is sufficiently smaller than the sound velocity *c*(*x*), Δ*c*(*x*) can be expressed as [20]

(2) $$∆c\left(x\right)=c\left(x\right)\frac{∆\tau }{∆t}.$$

Therefore, to obtain the change rate in sound velocity Δ*c*(*x*)/*c*(*x*) due to ultrasonic heating, Δ*τ*/Δ*t* was obtained by time-gating the acquired echo signal from the left lobe. However, since the echo shift time caused by ultrasonic heating is very small, it is important to calculate Δ*τ* with high accuracy. In this study, a method that combines two methods was used: the cross-correlation method, which can calculate the echo shift time even when the two signals contain different waveforms, and the autocorrelation method, which can calculate the echo shift time with high accuracy by using the phase information [26].

### 2.3. In Vivo Measurement of Change Rate in Sound Velocity

#### 2.3.1. Electrocardiogram (ECG)-Synchronized System

One challenge observed regarding in vivo measurement is the reduction in the number of valid data points due to suboptimal measurement conditions. This issue arises because the echo signals in the liver, which is the measurement target, is disrupted before and after heating, influenced by the heartbeat of anesthetized mice. In this study, data are considered valid if there is no disturbance in the echo signals in the liver before and after heating. To reduce the effects of this issue, we developed a system that acquires the electrocardiogram (ECG) of the mouse during measurement and synchronizes the timing of ultrasonic heating with the heartbeat of the mouse.

**Figure 3:**
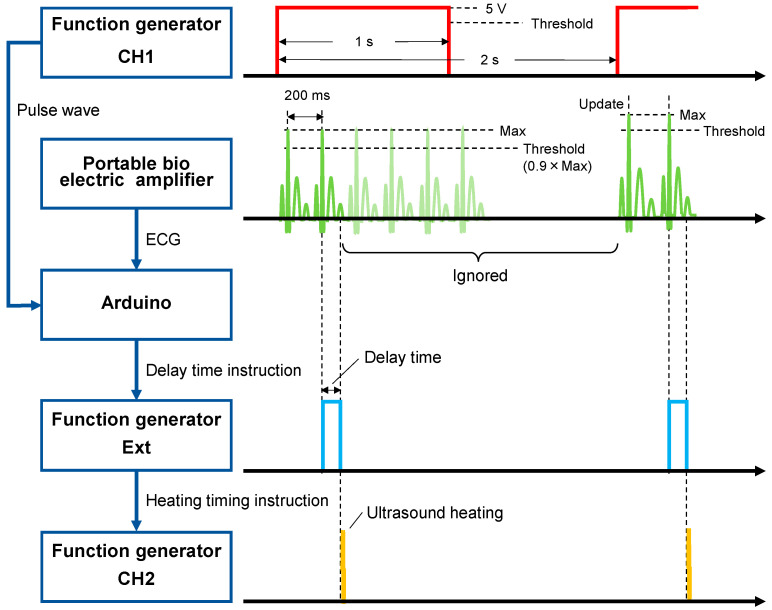
Experiment system and timing chart for in vitro measurement of change rate in sound velocity of the mouse livers due to the ultrasonic heating. The red, green, blue, and yellow lines represent the pulse wave for measurement timing control, the ECG signal, the delay time instruction signal, and the heating timing instruction signal, respectively.

Figure 3 shows the control chart of the ECG-synchronized system utilizing an Arduino. ECG signals can be recorded at any time by attaching electrodes to both forelimbs and the right hindlimb of the mouse. ECG was measured using a portable bioelectric amplifier (EBA-100, Unique Medical, Tokyo, Japan) set to a low cut-off frequency (LCF) of 1.5 Hz and a high cut-off frequency (HCF) of 240 Hz, and amplified by a factor of 10,000 during measurement. ECG waveforms were observed with an oscilloscope (MSO44B, Tektronix), and the interval from the R wave of the ECG until the waveform stabilized was measured and programmed into an Arduino as a delay time. A pulse wave with a frequency of 500 mHz and a voltage of 5 V was generated by a waveform generator (33500B, Agilent), and both the pulse wave and ECG signals were acquired using an Arduino. When the pulse wave turned on, the maximum ECG value was automatically obtained, and 0.9 times this maximum value was set as the threshold. When the condition that the pulse wave was turned on and ECG exceeded the threshold was met, a digital signal with a voltage of 5 V was output from the Arduino for the duration of the delay time. This digital signal was input into a waveform generator, initiating ultrasonic heating upon the falling edge of the signal. In this manner, the timing of ultrasonic heating is synchronized with the ECG. A characteristic of the system is that once the digital output is executed, ultrasonic heating is not triggered again until the next pulse wave, even if the condition is satisfied. The maximum ECG signals and threshold are automatically updated when the next pulse wave is turned on.

#### 2.3.2. Method of In Vivo Measurement

As a model for inducing steatotic liver disease, three 11-week-old BALB/c mice and two 17-week-old BALB/c mice were administered CDAHFD and standard water. One of the 11-week-old mice was treated for a duration of 1 week, while the remaining were treated for 4 weeks. The 17-week-old mice were treated for 7 weeks. As control groups, two 10-week-old BALB/c mice were provided with a standard diet and water. Under inhalation anesthesia, electrodes for ECG acquisition were attached to the forelimbs and right hindlimbs of the mice. Only the epidermis was incised to expose the peritoneum. The anatomical location and morphology of the liver were assessed using an ultrasonic diagnostic system (A9RK-00450, Konica Minolta, Tokyo, Japan). Following the measurement of the change rate in sound velocity, the livers were entirely removed for histopathological examination.

**Figure 4:**
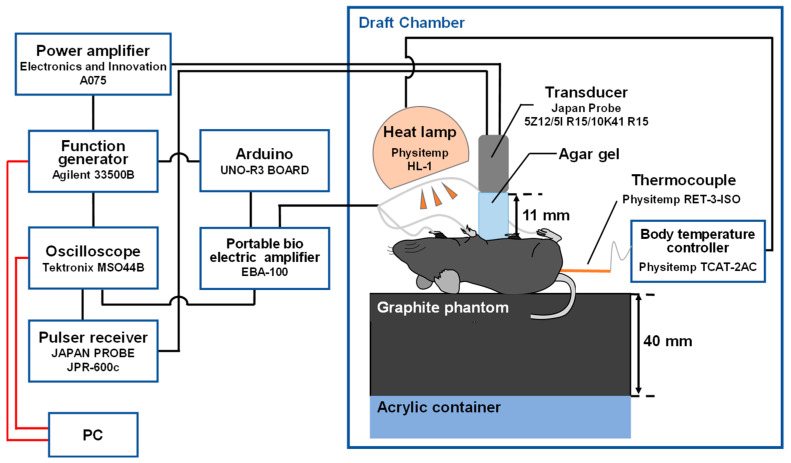
Experiment system for in vivo measurement of change rate in sound velocity of the mouse liver due to the ultrasonic heating. The red and black lines represent digital cables and analog cables, respectively.

Figure 4 shows the experimental system for in vivo measurement of the change rate in sound velocity of the mouse liver due to ultrasonic heating. Experiments were conducted in a draft chamber and controlled at approximately 37 °C with a heat lamp to prevent the mice from decreasing body temperature due to anesthesia. Mice with epidermis-only incisions were positioned supine on an agar-gel block mixed with graphite powder. Dispersion of graphite powder in the agar-gel block attenuated ultrasonic waves transmitted from the back of the mouse to the agar-gel block and eliminated the effect of reflected waves from the boundary between the acrylic block and the agar-gel block on the measurement.

For in vivo measurements of change rate in sound velocity, concave circular and concave ring-shaped transducers with the same shape as for in vitro measurements were used. The concave circular transducers for pulse-echo methods used in this experiment were one with a center frequency of 8.3 MHz and a focal length of 14.8 mm and another with a center frequency of 9.6 MHz and a focal length of 14.1 mm. The center frequency of both concave ring-shaped transducers for ultrasonic heating was 5 MHz. Agar-gel block cut approximately 11 mm thickness was placed between the transducer and the mouse so that the transducer was focused on the mouse liver. The function generator, power amplifier, pulsar receiver, and oscilloscope were connected as in the in vitro measurements. In addition, the ECG-synchronized system shown in Figure 3 was connected to the function generator. The ECG signal was also observed by the oscilloscope. Mouse livers were exposed to ultrasound of 1.6 MPa in negative sound pressure for 10 ms or 1.1 MPa in negative sound pressure for 20 ms for ultrasonic heating. The ultrasonic heating and echo signal acquisition were repeated 20 times at 2 s intervals. The change rates in sound velocity Δ*c*(*x*)/*c*(*x*) due to ultrasonic heating were obtained in the same manner as the in vitro measurements.

After the in vivo measurement of the change rate in sound velocity, histopathological examination was performed on the excised livers, and the lipid accumulation rate was calculated by quantitative image analysis of hematoxylin-eosin (HE)-stained images. The lipid accumulation rate is defined as the ratio of lipid droplets to images. The HE-stained images were grayscaled, binarized, and hole-filled. The lipid droplets in the image were extracted as circular objects and their areas were calculated. Because each image includes blood vessels, only the blood vessels were extracted from the image, and their area was also calculated. The lipid accumulation is given by

(3) $$F=\frac{S_{L}}{S-S_{V}}\times 100$$

using the area of lipid droplets *S*_L_, the area of image *S*, and the area of blood vessels *S*_V_.

## 3. Results

### 3.1. TMM Experiments

**Figure 5:**
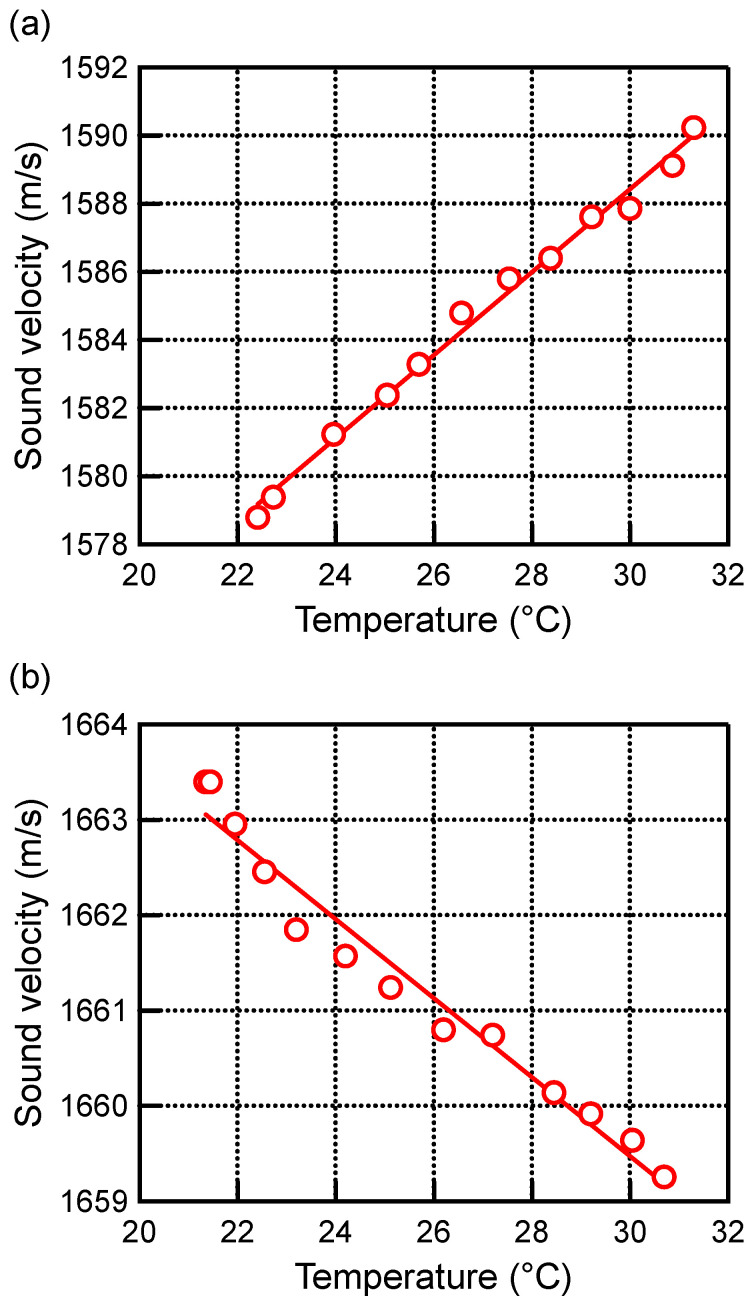
Sound velocities of TMM at (**a**) 20.00% and (**b**) 43.00% glycerin concentration as a function of temperature. The red line shows the result of the liner approximation.

**Figure 6:**
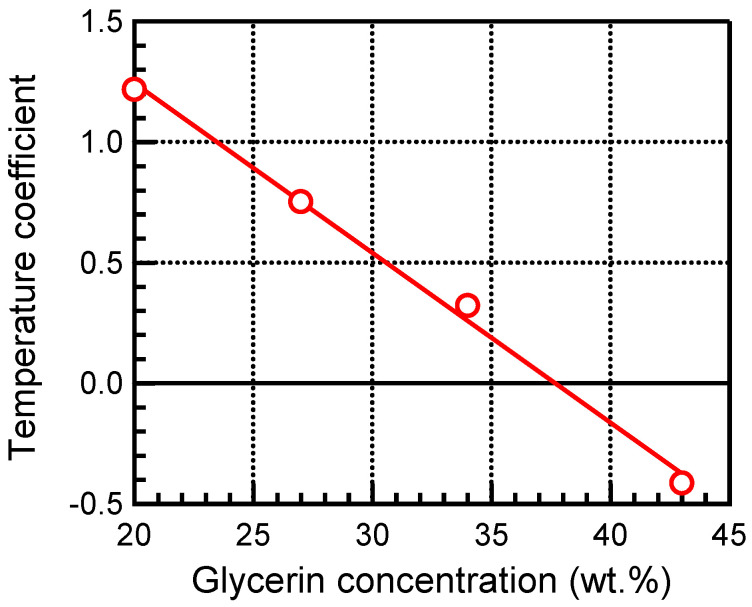
Temperature coefficients of sound velocity for each glycerin concentration of TMM. The red line shows the result of the liner approximation.

Figure 5 shows the sound velocity measurements of TMM at 20.00% and 43.00% glycerin concentration as a function of temperature. As shown in Figure 5a, the sound velocity of TMM with 20.00% glycerin concentration increased with increasing temperature. A linear approximation of these results yielded a temperature coefficient of 1.22 ± 0.04 m/s/°C. On the other hand, for TMM with a glycerin concentration of 43.00%, the sound velocity decreased with increasing temperature as shown in Figure 5b. A linear approximation was used to obtain the temperature coefficient, which was −0.41 ± 0.03 m/s/°C. Similarly, the temperature coefficients for TMM at 27.00% and 34.00% glycerin concentration were determined. The temperature coefficients for each glycerin concentration of TMM are shown in Figure 6. Error bars for each temperature coefficient by linear approximation were less than 0.04 m/s/°C.

### 3.2. In Vitro Experiments of Mouse Livers

**Figure 7:**
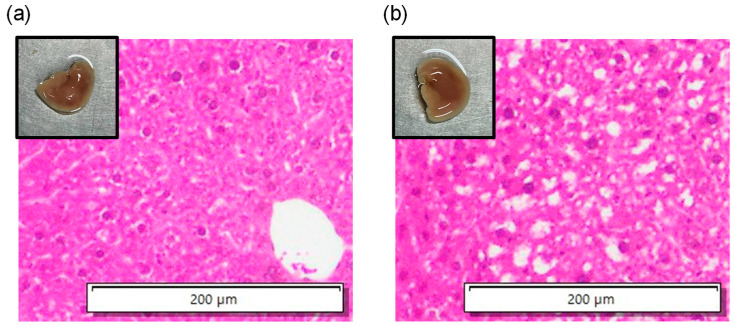
Photographic images and HE-stained images in the histopathological analyses in the left lobe of the liver of 6-week-old (**a**) control and (**b**) 3-day CDAHFD mice.

Figure 7 shows photographic images and HE-stained images in the histopathological analyses in the left lobe of the liver of 6-week-old control and 3-day CDAHFD mice. HE-stained images showed lipid droplets and ballooning in mice in the CDAHFD group. Thus, the liver contained more fat in the CDAHFD group than in the control group; moreover, steatotic liver disease was induced in mice in the CDAHFD group.

**Figure 8:**
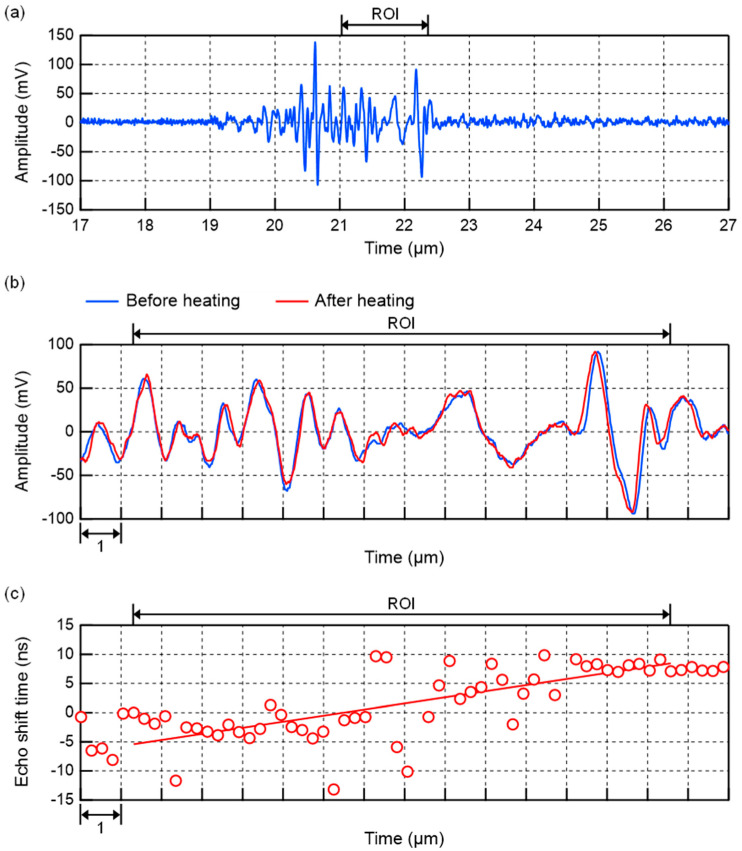
Example of ultrasound echo signals acquired in the left lobe of the liver of 6-week-old control group mice. (**a**) Echo signal of before heating. (**b**) Echo signals of before and after heating near the ROI. (**c**) Calculation results of echo shift time after heating. The red line shows the result of the linear approximation. The slope of the line shows the change rate in sound velocity.

**Figure 9:**
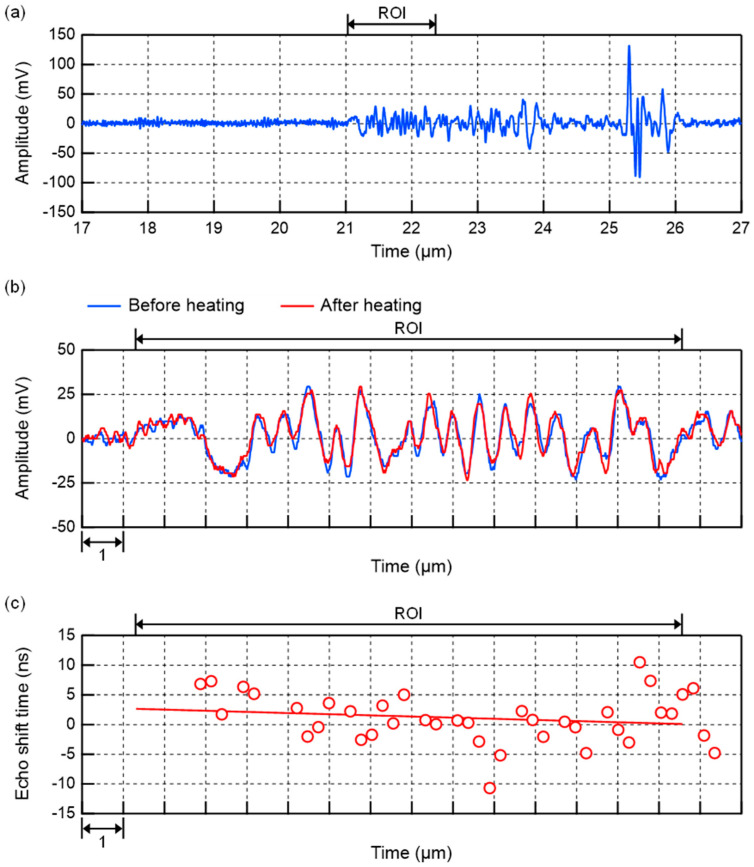
Example of ultrasound echo signals acquired in the left lobe of the liver of 6-week-old steatotic liver group mice. (**a**) Echo signal of before heating. (**b**) Echo signals of before and after heating near the ROI. (**c**) Calculation results of echo shift time after heating. The red line shows the result of the linear approximation. The slope of the line shows the change rate in sound velocity.

**Figure 10:**
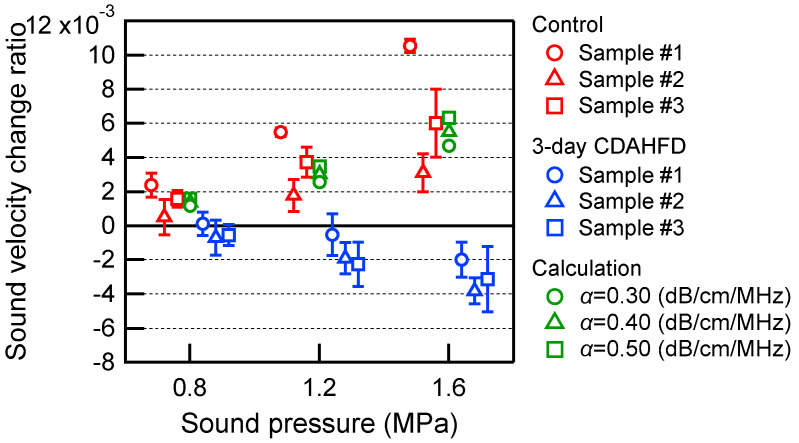
Change rates in sound velocity due to the ultrasonic heating in the left lobe of the liver of 6-week-old mice. The red plots represent the control group, and the blue plots represent the 3-day CDAHFD group. The solid lines represent error bars as the standard deviation obtained from ten measurements of the same liver sample. The green plots show the simulation results for a liver calculated with varying values of acoustic attenuation α.

**Figure 11:**
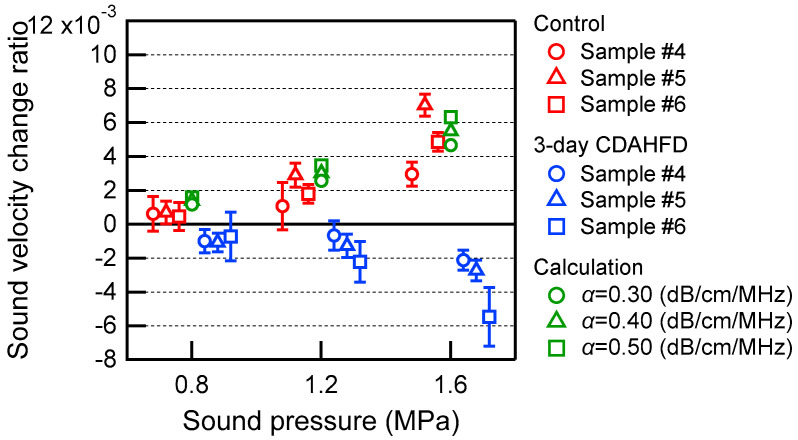
Change rates in sound velocity due to the ultrasonic heating in the left lobe of the liver of 12- to 14-week-old mice. The red plots represent the control group, and the blue plots represent the 3-day CDAHFD group. The solid lines represent error bars as the standard deviation obtained from ten measurements of the same liver sample. The green plots show the simulation results for a liver calculated with varying values of acoustic attenuation α.

Figure 8 shows an example of ultrasound echo signals acquired in the left lobe of the liver of 6-week-old control group mice. Since the focus of the transducer used for heating in this experiment was 15.5 mm, the region of interest (ROI) was set at 21.1–22.4 μs in Figure 8a. Figure 8b shows a magnified image of the echo signals in the ROI before and after ultrasonic heating. The echo shift time after heating was then calculated from these two echo signals, as shown in Figure 8c. Figure 8c also shows the approximate line obtained from this echo shift time. As shown in Equation (2), the slope of this line is the change rate in sound velocity Δ*c*(*x*)/*c*(*x*) due to ultrasonic heating. In this result, the value of the change rate in sound velocity was positive. In the same manner, Figure 9 shows an example of ultrasound echo signals acquired in the left lobe of the liver of 6-week-old steatotic liver group mice. The results of the linear approximation in Figure 9c show that the value of the change rate in sound velocity is negative. These results of the change rate in sound velocity are plotted in Figure 10 for each sound pressure. As a comparison, the simulation results for a liver calculated with varying values of acoustic attenuation α are also shown. The results for 12- to 14-week-old C57BL/6 mice were calculated in the same manner for the change rate in sound velocity and plotted in Figure 11. The error bars in Figure 10 and Figure 11 represent the standard deviation obtained from ten measurements of the same liver sample.

### 3.3. In Vivo Experiments of Mouse Livers

**Figure 12:**
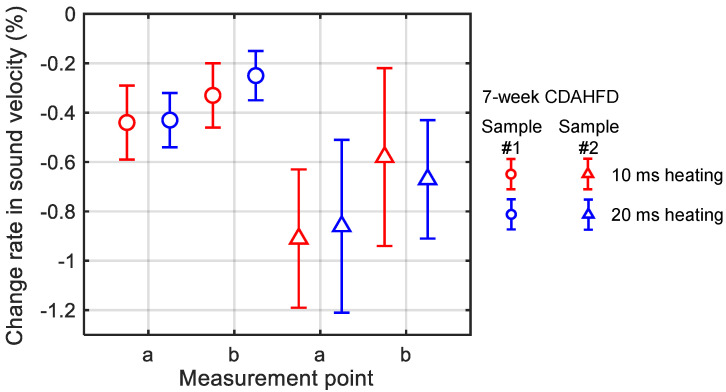
Change rates in sound velocity due to the ultrasonic heating in the mouse livers of the 7-week CDAHFD group in in vivo measurement. The red and blue plots represent heating times of 10 ms and 20 ms with corresponding sound pressures of 1.6 MPa and 1.1 MPa, respectively. Measurement point a and b represent two measurement points on the same mouse. The solid lines represent error bars as the standard deviation obtained from twenty measurements at the same measurement point.

Echo signals from the mouse livers were acquired in vivo using the ECG-synchronized system. The change rate in sound velocity Δ*c*(*x*)/*c*(*x*) due to ultrasonic heating was then calculated as in the in vitro measurements. In the 7-week CDAHFD group, the ultrasonic heating time was set to 10 ms with a sound pressure of 1.6 MPa or the ultrasonic heating time was set to 20 ms with a sound pressure to 1.1 MPa, in order to investigate the measurement conditions. The results of these change rates in sound velocity are shown in Figure 12. The error bars in Figure 12 represent the standard deviation obtained from twenty measurements at the same measurement point.

**Figure 13:**
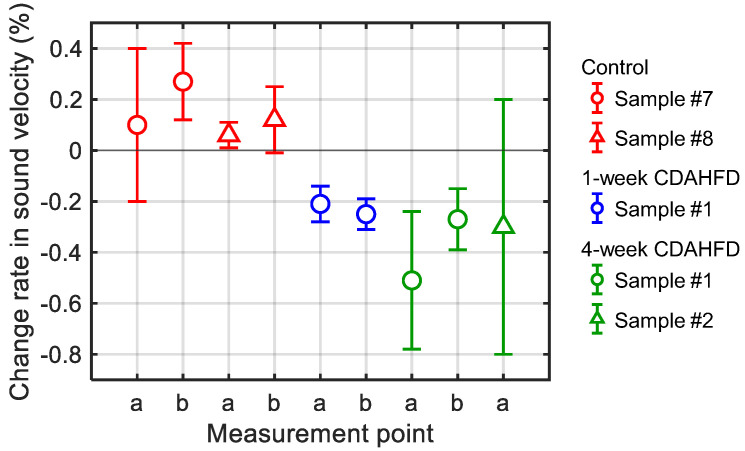
Change rates in sound velocity due to the ultrasonic heating in the mouse livers in in vivo measurement. The red plots represent the control group, the blue plots represent the 1-week CDAHFD group, and the green plots represent the 4-week CDAHFD group. Measurement point a and b represent two measurement points on the same mouse. The solid lines represent error bars as the standard deviation obtained from twenty measurements at the same measurement point.

**Figure 14:**
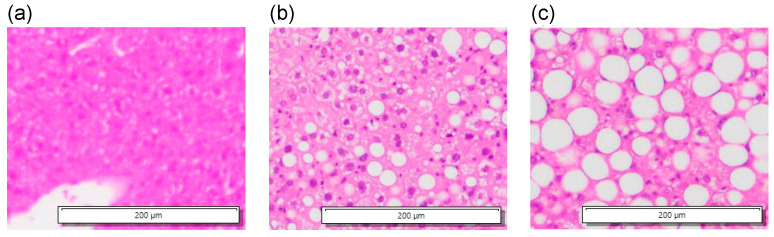
HE-stained images in the histopathological analyses in the mouse liver of (**a**) control, (**b**) 1-week CDAHFD, and (**c**) 4-week CDAHFD group.

**Figure 15:**
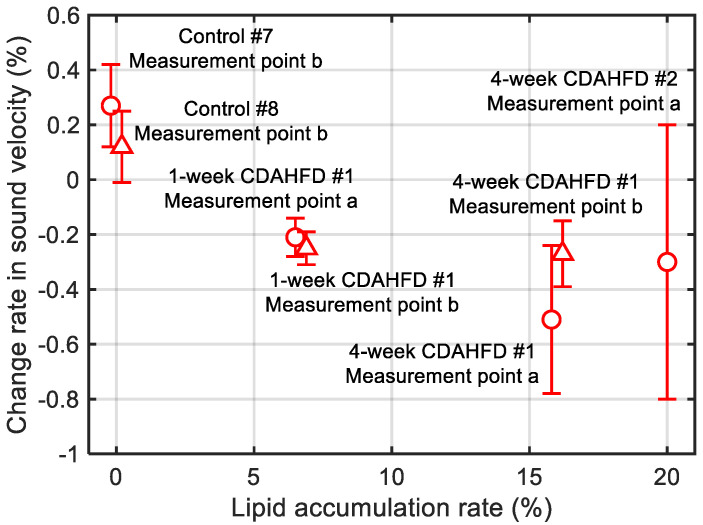
Change rate in sound velocity due to the ultrasonic heating of the mouse livers as a function of the lipid accumulation rate. Measurement point a and b represent two measurement points on the same mouse. The solid lines represent error bars as the standard deviation obtained from twenty measurements at the same measurement point.

Figure 13 shows the in vivo measurement results of the change rates in sound velocity due to the ultrasonic heating in the mouse livers of the control, the 1-week CDAHFD, and the 4-week CDAHFD groups. The error bars in Figure 13 represent the standard deviation obtained from twenty measurements at the same measurement point. Their HE-stained images are shown in Figure 14. HE-stained images of the CDAHFD group in Figure 14b,c showed lipid droplets, indicating the presence of macrovesicular steatosis in the mouse liver. In addition, enlarged lipid droplets were observed in the HE-stained images of the 4-week CDAHFD group compared to the 1-week CDAHFD group, along with hepatocyte ballooning and lobular inflammation. Thus, the lipid accumulation was more prominent in the liver of the CDAHFD group than in the control group, steatotic liver disease was induced in mice in the CDAHFD group. Figure 15 shows the change rate in sound velocity due to the ultrasonic heating of the mouse livers as a function of the lipid accumulation rate calculated by analyzing the HE-stained images.

## 4. Discussion

Figure 6 shows that the temperature coefficient of sound velocity decreases with increasing glycerin concentration in TMM and turns from a positive value to a negative value. Water has a positive temperature coefficient, and glycerin has a negative coefficient. Therefore, as the glycerin concentration is increased, the temperature coefficient of TMM approaches that of glycerin. Fat tissue has also been found to have a negative temperature coefficient. These results suggest that the temperature coefficient changes from positive to negative depending on the degree of fat accumulation in the non-fat tissues. In addition, the temperature coefficient of TMM was 0 m/s/°C when the glycerin concentration was 37.5% in this result. Therefore, based on the results of Figure 6, it is possible to investigate the method of measuring the various change rates of sound velocity from positive to negative using TMM. In this study, the glycerol concentration of the TMM was varied to simulate fat accumulation. To more appropriately model the effects of different pathological stages, such as steatosis and fibrosis, further investigation is necessary by varying the scattering agents and their density and size distribution within the TMM.

From Figure 10 and Figure 11, the change rates of sound velocity by ultrasonic heating were measured in the left lobe of the mouse livers by the proposed method, resulting in positive values in the control group and negative values in the steatotic liver group. In the control group, the change rates of sound velocity were increased by increasing the ultrasound pressure for heating. This indicates that the increase in sound pressure heats the liver more and increases the sound velocity more. The same trend was also observed in the calculation results, suggesting that the liver was indeed heated in the experimental system shown in Figure 2. On the other hand, in the steatotic liver group, the change rates of sound velocity were negatively increased by increasing the ultrasound pressure for heating. When the sound pressure was increased up to 1.6 MPa, the difference between the control group and the steatotic liver group increased, and a positive/negative distinction could be observed. However, there were also results with large error bars for each individual measurement. This may be because the echo signal was not well obtained in the ROI, which was set near the focus of the ultrasound for heating, due to the curvature of the liver surface.

Figure 12 shows that there were no significant differences in the change rates of sound velocity between the conditions of a 10 ms heating time with a sound pressure of 1.6 MPa and the conditions of a 20 ms heating time with a sound pressure of 1.1 MPa. Therefore, the ultrasonic heating by these two conditions was at the same level. Because the lower sound pressure is assumed to have a lower physiological load on the mice, the conditions of a 20 ms heating time with a sound pressure of 1.1 MPa were used in the subsequent in vivo measurements.

The change rates in sound velocity by ultrasonic heating were measured in vivo in the mouse livers by the proposed method, resulting in positive values in the control group and negative values in the steatotic liver group, similar to the results of in vitro measurement as shown in Figure 13. On the other hand, there was no significant difference in the change rates of sound velocity by ultrasonic heating between the 1-week CDAHFD group and the 4-week CDAHFD group. In addition, the measurement results of the 4-week CDAHFD group showed more variability and less valid data in the twenty measurements compared to that of the 1-week CDAHFD group. This may be due to the progression of lipid accumulation, which could have attenuated the echo signal from the liver. Instead of analyzing only about the center frequency of the transducer for the pulse echo measurement, it is also necessary to investigate the frequency components with large amplitude near the center frequency to reduce the variation. From Figure 15, the change rate in sound velocity increased in a negative direction with increasing the lipid accumulation rate in the mouse liver. However, for the reasons discussed above, there were the results with large error bars, especially when the lipid accumulation rate was large. Therefore, further study of in vivo measurement systems to reduce measurement variation is needed to estimate lipid accumulation rate by the change rate of sound velocity.

## 5. Conclusions

In this study, an ultrasonic diagnostic method for steatotic liver disease based on the temperature dependence of the sound velocity is experimentally discussed. As a preliminary experiment before considering steatotic liver evaluation, temperature coefficients of sound velocity of TMM with glycerol concentration were measured. As a result, the temperature coefficient of TMM decreased as the glycerol concentration increased. It changed from positive value to negative value at 37.5% of glycerol concentration. Thus, it is expected that the temperature coefficient of sound velocity of liver decreases as lipid accumulation rate increased. The change rates in sound velocity by ultrasonic heating were measured in vitro on the left lobe of the liver of mice with steatotic liver induced by CDAHFD. Heating the left lobe of the liver with ultrasound resulted in a negative rate of sound velocity change in the steatotic liver group, whereas the control group showed a positive rate of sound velocity change. Furthermore, in vivo measurements of the mouse liver using an ECG-synchronized system were then demonstrated. The results of the change rates in sound velocity were positive in the control group and negative in the steatotic liver group, similar to the in vitro measurements. Thermophysical properties of mouse liver tissue can be measured to determine whether the liver is normal or steatotic. The change rate in sound velocity tended to increase negatively with increasing lipid accumulation rate, while the measurement variation also increased. By reducing the variation in in vivo measurements through improvements such as increasing the echo signal intensity from the liver, it is expected that the lipid accumulation rate can be estimated from the change rate in sound velocity.

## Data Availability

The original contributions presented in this study are included in the article. Further inquiries can be directed to the corresponding author.

## Abbreviations

The following abbreviations are used in this manuscript:

HCC	Hepatocellular carcinoma
TMM	Tissue-mimicking material
CDAHFD	Choline-deficient, L-amino acid-defined, and high-fat diet
ECG	Electrocardiogram
LCF	Low cut-off frequency
HCF	How cut-off frequency
HE	Hematoxylin-eosin
ROI	Region of interest
